# A questionnaire survey on diseases and problems affecting sheep and goats in communal farming regions of the Eastern Cape province, South Africa

**DOI:** 10.4102/jsava.v87i1.1348

**Published:** 2016-08-31

**Authors:** Gareth F. Bath, Mary-Louise Penrith, Rhoda Leask

**Affiliations:** 1 Department of Production Animal Studies, University of Pretoria, South Africa; 2 Department of Veterinary Tropical Diseases, University of Pretoria, South Africa

## Abstract

A questionnaire of 15 questions was completed by four categories of respondents with the aim of establishing the experience and opinions of these groups on the constraints including animal health problems for communal, small-scale sheep and goat farming in the Eastern Cape province of South Africa. The questionnaires were completed independently and categories were representative of the areas investigated. Analysis of responses was done by means, ranges, votes and clusters of responses. Comparisons between the responses of the four categories were made to identify similarities or contrasts. The results revealed that of non-veterinary concerns, stock theft was the major problem for these farms. Nutrition was a further major constraint. A third area of significant concern was the provision or availability of facilities like fences, water troughs, dips and sheds. Lack of marketing and business skills were also seen as important deficiencies to be rectified so as to promote profitable farming. Of the most important veterinary problems identified, the provision, availability, cost and care of drugs and vaccines were seen as major stumbling blocks to effective disease control, as well as lack of access to veterinary services. The most important diseases that constrain small-ruminant livestock farming in the farming systems investigated were sheep scab and other ectoparasites, heart water, enterotoxaemia, internal parasites and bluetongue. A lack of knowledge in key areas of small-stock farming was revealed and should be rectified by an effective training and support programme to improve the contribution of small-ruminant farming to livelihoods in these communities.

## Introduction

A questionnaire survey was undertaken among four interest groups as part of a project to improve livelihoods and create wealth through smallholder livestock production in the Eastern Cape province of South Africa. The aim was to determine the most important constraints experienced in small-scale ruminant production. Due to their cultural value in these communities, trading in cattle is less commonly practised than trading in sheep and goats, and for this reason, the study concentrated on the latter species.

The opinions and experiences of people actively involved in identifying and dealing with livestock farming health issues have too often been ignored or deprecated as being of little or no value, on the basis that this information is not sourced from scientific experiments or diagnostic tests (Sackett *et al*. 1996). However, there is a growing realisation that much valuable and sometimes unique information can be easily and fairly reliably obtained using structured questionnaires or other systems to elicit the experiences of those involved (Sackett *et al*. 1996). This knowledge is of particular value when sourced from independent, scientifically trained groups, but it is also of importance to compare these views with those closest to the problem even though they may have little or no scientific background. In some published articles, investigators report only on the experiences and opinions of farmers and communities (Makgatho, McCrindle & Owen 2005). While such information also has value, it would be greatly enhanced if it were compared and contrasted with the views of other groups more independently objective and scientifically trained (Sackett *et al*. 1996). A prerequisite for small-scale farmers to succeed is that herd or flock health must be promoted, because animal diseases in the broadest sense if left undiagnosed and unchecked will negate all efforts to improve livestock farming for these communities (Masiko & Mafu 2004; Rumosa Gwaze, Chimonyo & Dzama 2009; Sebei, McCrindle & Webb 2004).

Obtaining the experiences and views of interest groups best placed to express valid inputs on livestock health in communal small-scale farming areas helps to identify successes as well as failures and indicates where more concerted action is required to rectify deficiencies (Bembridge & Tapson 1993). The objective of this survey of experience and opinion was to provide information to help provincial authorities to prioritise actions and supply the maximum benefit to target communities.

## Materials and methods

### Questionnaires

The questionnaire design was based on principles previously tested and described (Bailey 1978; Berdie 1973; Berdie, Anderson & Niebuhr 1986; Geer 1988; McClendon & O’Brien 1988; Montgomery & Crittenden 1977; Sheatsley 1983).

The questions were formulated to elicit key information and then tested on a small group of veterinarians and animal health technicians for clarity and usefulness before modification and inclusion in the questionnaire. The questions were grouped in three sections, the first four (A–D) being overall viewpoints, the next two (E and F) concentrated on general animal disease and the last nine (G–O) dealt with sheep and goat health. Thus, there were 15 questions to be answered. Most questions (A, C, E, F, G, H, I, K, L and N) included a rating scale (0–10) to enable comparison and establish rankings. Question D was a category marking while J required three possible rankings. Questions B, F, J, K, M and O were partly or largely open-ended. For ease of reference, the list of questions is available as Appendix 1.

The findings of the questionnaires, after analysis, were used as the basis for most of the training material generated and provided.

### Participants

Four important groups involved in farming livestock and in animal health, namely communities of livestock farmers, their livestock production advisors, animal health technicians and veterinarians were identified. The aim was to establish the degree of agreement among these groups on a range of selected topics, the importance rating of each aspect and solutions used or required. Where possible, reasons for significant disparities between groups were analysed.

The veterinarians and animal health technicians were from the entire Eastern Cape province, including commercial regions, but especially the communal areas, while production advisors only serviced the communal areas. Communities were identified for participation by a random allocation of all communal regions but based on representation of all farming and climatological types. The four communal regions designated 20, 21, 23 and 24 in the results were identified by the National Wool Growers’ Association as groupings of communal farming areas in municipal districts in the Eastern Cape. Each one is regarded as reasonably homogeneous and together represent all the districts in this study. These allocations were based on previous surveys for other purposes (Tapson 2004).

The size of farming community groups (*n* = 40) varied from 4 to 22 (average 10.5) farmers or owners distributed throughout the survey region. There were 9 farm advisors paid by or seconded to the National Wool Growers’ Association and 1 supervisor, 30 animal health technicians and 28 veterinarians.

All participants were asked to answer the questionnaire in accordance with their own experience and knowledge. Any points requiring clarification or explanation were dealt with during the time when respondents gave their answers to the questions. In the case of production advisors, animal health technicians and veterinarians, the survey was conducted simultaneously during an annual meeting of Eastern Cape provincial veterinary staff. For farming communities, the surveys were conducted individually at each site by an experienced survey interviewer in the local vernacular (isiXhosa). All interviews for this segment were conducted by explaining the question, then asking by show of hands which response was regarded as the correct answer. These responses were then recorded by the interviewer.

Since veterinarians, animal health technicians and production advisors all completed their questionnaires on their own at the same time, there was no possibility of discussion, caucusing or mutual influence. Thus, the results can be seen as genuine, unbiased opinions. Farmers were interviewed in groups (communities), so peer influence could and probably did play a role in responses given. However, any community’s responses to questions were very unlikely to have been influenced by other communities.

### Analysis

Questions were analysed by group category and where possible along similar lines (apart from the open-ended questions).

All responses from all groups were collected, checked and filed in groups according to the categories of respondents. Because it was difficult to allocate veterinarians to the categories originally envisaged, the responses of all veterinarians including those in management positions were pooled for analysis. Similarly, the responses of all animal health technicians of any rank were pooled, including those who styled themselves as animal production advisors but were not part of the National Wool Growers’ Association team, who were clearly identified and analysed separately.

Analysis of questions A, C, E, F, G, H, I, L and N was simply by calculating mean and range per answer per group. Questions D, K and J were recorded by the number of votes for various categories per group, and the answers to the more open-ended questions B, J, M and O and partially F and K were analysed by clusters of similar responses.

## Results

### General issues related to livestock farming

There was general agreement among the groups that agriculture and ruminant livestock were important for livelihoods, with overall high averages, although with wide ranges (Table 1).

**TABLE 1:** Importance of agriculture to the standard of living.

Subcategory	Farmers		Advisors		Technicians		Veterinarians	
	Mean	Range	Mean	Range	Mean	Range	Mean	Range
Agriculture in general	9.8	5–10	8.1	6–10	8.7	4–10	8.5	1–10
All forms of livestock	10.0	10–10	6.8	0–9	7.4	0–10	8.3	4–10
Sheep	10.0	9–10	7.9	7–9	7.3	2–10	7.9	3–10
Goats	10.0	10–10	6.2	4–8	7.0	0–10	6.9	2–10
Cattle	9.8	5–10	7.4	6–9	8.0	5–10	7.8	1–10
Other (chickens, pigs)	4.7	0–10	4.8	0–8	5.9	0–10	5.6	2–10

Scale 1–10 from least to most important.

Tables 2 and 3 reflect the constraints and difficulties according to four or more responses among the farmer groups and three or more responses among the technical groups respectively. Minority responses covered a wide range of additional problems involving issues from lack of infrastructure, resources, support and inputs to poor financial returns, lack of young farmers and climate and invasive plants. These issues were included in the project report for consideration by the Eastern Cape authorities (G.F. Bath, 2013, unpublished report to Wellcome Foundation) but are not detailed here.

**TABLE 2:** Difficulties and constraints: Those with four or more responses communities of farmers.

Problem	Region				Total	%
	20	21	23	24				
Stock theft	11	8	5	10	34	13.3
Drought and lack of drought relief	7	7	7	9	30	11.8
Fencing	6	8	7	8	29	11.4
Lack of water supply or dams	11	1	5	3	20	7.8
Shortage of dipping tanks especially for small stock	6	2	6	4	18	7.1
Cost of drugs	-	3	3	8	14	5.5
Lack of shearing shed	6	1	3	4	14	5.5
Lack of farmer knowledge about livestock disease control	4	-	-	9	13	5.1
Livestock disease	-	5	7	-	12	4.7
Sheep scab	3	1	1	6	11	4.3
Lamb losses	2	3	-	2	7	2.7
Veld fires	-	3	2	2	7	2.7
Drug availability	3	-	1	-	4	1.6
Disease control	4	-	-	-	4	1.6
No livestock guards or rangers	-	2	2	-	4	1.6
**Number (%) of responses**	**-**	**-**	**-**	**-**	**221**	**86.7**

It is worth noting that, in contrast to the farming communities, the advisors, technicians and veterinarians emphasised lack of capacity on the part of the farmers compounded by lack of education, training and information as the major constraint for profitable farming (Table 3). This was also reflected in the questions relating specifically to constraints affecting small-ruminant stock farming as opposed to livestock farming in general (Table 4). Among the three most important issues according to each of the groups, only veld management was common to all the groups. Training and knowledge of farmers was again common to the three technical groups, while for farmers, it was the fourth ranked priority. Farmers and veterinarians rated the provincial budget among their three most important issues, while farmers and their advisors placed fences among their top three priorities. Only technicians rated facilities for farmers among their top three issues, while only the advisors rated stock theft among their top three priorities. The farmers gave fairly high priority rankings to all the issues covered, with animal identification being the lowest. Only the advisors accorded a fairly high priority to animal identification. Predation (predators or dogs) was considered the lowest priority by all the technical groups, while it was ranked sixth by the farmers.

**TABLE 3:** Production advisors, animal health technicians, veterinarians.

Problem	Advisors	Technicians	Veterinarians	Total	%
Education or information or training	2	14	15	31	16.2
Infrastructure (fencing, housing, transport)	8	7	6	21	11.0
Veld or nutritional management	4	7	7	18	9.4
Resources (grazing land, genetic stock, skilled labour)	4	5	7	16	8.4
Traditional farming practises	1	7	6	14	7.3
Finances	1	3	10	14	7.3
Market access and marketing skills	-	4	7	11	5.8
General livestock management	2	4	3	9	4.7
Lack of farmer cooperation and dependence on hand-outs	-	4	4	8	4.2
Poor government staffing and service	1	3	2	6	3.1
Drug unavailability or inappropriate drug use	1	2	2	5	2.6
Stock theft	3	2	-	5	2.6
Biosecurity practises	1	1	2	4	2.1
Lack of government involvement and support	-	2	1	3	1.6
Livestock disease	2	1	-	3	1.6
No primary healthcare or prevention	1	-	2	3	1.6
Breeding management	-	1	2	3	1.6
**Number (%) of responses**	**-**	**-**	**-**	**174**	**91.1**

The majority of farmers and advisors believed that more than 50% of the annual provincial agricultural budget should be spent on addressing the constraints identified, while the majority of technicians agreed that at least 30% to more than 50% should be allocated and the majority of veterinarians voted for between 20% and 50%.

**TABLE 4:** Major constraints to profitable small-stock farming.

Subcategory	Farmers		Advisors		Technicians		Veterinarians	
	Mean	Range	Mean	Range	Mean	Range	Mean	Range
Budget provincial department	9.6	4–10	5.8	0–9	6.2	0–10	7.4	0–10
Staff provincial department	7.7	0–10	4.7	0–8	6.0	0–10	6.6	0–10
Facilities for farmers	7.9	0–10	7.2	4–9	7.0	0–10	6.5	0–10
Training of farmers (knowledge)	9.3	4–10	7.9	5–10	7.2	0–10	8.6	5–10
Drug supply chain	8.0	0–10	6.8	4–9	5.7	0–10	5.9	0–10
Marketing chain	9.1	0–10	6.8	4–10	5.9	0–10	5.5	0–10
Fences	9.9	7–10	8.8	5–10	6.3	0–10	6.5	0–10
Veld management	9.6	3–10	8.7	6–10	6.7	0–10	8.3	4–10
Stock theft	8.8	3–10	7.9	4–10	6.2	0–10	5.0	0–10
Transport allocations	8.2	0–10	6.0	3–8	5.1	0–10	4.1	0–9
Predators or dogs	8.9	0–10	5.3	0–9	3.8	0–10	3.5	0–10
Animal identification	6.9	0–10	7.4	4–10	5.6	0–10	4.8	0–10
Other	0.3	0–10	1.1	0–2	0.8	0–9	2.0	0–10

### Animal health issues for sheep and goat farming

Although the Part II questions covered livestock in general, the categories of disease are all applicable to small ruminants (Table 5) and all four groups agreed independently upon six diseases of small ruminants as the most important (Table 6). Therefore, the results of Parts II and III are presented together and diseases of livestock other than small ruminants mentioned by a minority of respondents are omitted.

**TABLE 5:** Importance of livestock disease categories.

Subcategory	Farmers		Advisors		Technicians		Veterinarians	
	Mean	Range	Mean	Range	Mean	Range	Mean	Range
Parasites: Internal	9.7	5–10	8.3	5–10	7.6	0–10	8.2	3–10
Parasites: External	9.7	4–10	7.7	5–10	7.4	1–10	8.1	3–10
Infections	9.5	5–10	7.1	5–8	5.6	0–9	7.6	4–10
Toxic plants	8.2	0–10	3.3	0–5	3.7	0–6	3.7	0–7
Nutrition	8.8	0–10	8.0	6–10	7.9	4–10	8.3	0–10
Other	0.0	0–0	0.0	0–0	0.7	0–7	0.0	0–0

Parasites (both internal and external) and nutrition emerged as important disease categories, but only parasites were accorded high importance by all four groups. The technical groups considered nutrition to be highly important, with the technicians and veterinarians ranking it as most important, whereas the farmers rated it second last, behind parasites and infectious diseases. Toxic plants were rated lowest of the five categories by all four groups, although the farmers accorded relatively high ratings to all five categories (Table 5).

The second question of Part II (F), on animal disease in general, was largely open-ended and received inconsistent responses, but all groups agreed on the diseases listed in Table 6 as the most important. These results were repeated in question J, specific to sheep and goats. All four groups agreed that diseases were important in small-ruminant farming, with farmers awarding a score of 10 out of 10 and the three technical groups awarding scores ranging from 7 to 7.3 out of 10. The six diseases or conditions that emerged as most important were sheep scab, heartwater, clostridial diseases (mainly pulpy kidney), endoparasites, bluetongue and ectoparasites other than sheep scab mite, in that order. Sheep scab was ranked as the highest priority by the farmers, advisors and technicians and as a high priority by veterinarians. Pulpy kidney, bluetongue and worms were ranked as important by all the groups. Heartwater was ranked as important by the three technical groups. The farmers additionally identified pasteurellosis, *Coenurus cerebralis* (‘gid’) and black quarter as among the most important diseases. Veterinarians considered malnutrition to be an important underlying factor for most of the disease conditions.

**TABLE 6:** Summary of responses listing the top six livestock disease problems.

Problem or disease	Category of respondents				
	Farmers	Advisors	Technicians	Veterinarians	Mean score
Sheep scab	1	1	1	1	1.00
Heartwater	2	2	3	4	2.75
Clostridial diseases	3	3	4	3	3.25
Internal parasites	4	6	6	2	4.50
Bluetongue	5	4	5	5	4.75
Other ectoparasites	6	5	2	6	4.75

Ranking of importance: 1 (most) to 6 (least) of the top six problems for control.

In relation to production parameters (Table 7), the number of lambs born emerged as the most important in the opinion of all four groups.

**TABLE 7:** Importance of production categories.

Subcategory	Farmers		Advisors		Technicians		Veterinarians	
	Mean	Range	Mean	Range	Mean	Range	Mean	Range
Reproduction or number of lambs or kids born	9.5	5–10	8.4	6–10	7.7	0–10	8.2	0–10
Number of sheep sold per year	8.2	4–10	7.0	5–9	6.4	0–10	6.9	0–10
Number of goats sold per year	8.2	4–10	6.2	59	5.9	0–10	6.0	0–10
Amount of wool sold per year	8.3	2–10	8.3	6–10	7.5	0–10	6.7	0–10
Quality of wool sold	8.5	3–10	8.1	6–10	7.2	0–10	6.7	0–10
Others (specify income source)	0.0	0–0	1.9	0–10	0.4	0–8	0.0	0–0

Question I provided a list of syndromes to be rated according to their importance (Table 8). The farmer scores all fell between 8.6 and 9.9 and were therefore somewhat uninformative, but lambing problems and not eating received some of the highest scores. The advisors and the veterinarians gave the highest scores to sudden deaths and diarrhoea in lambs and skin problems in adults, with the addition of loss of body condition in weaners by the veterinarians; the technicians rated skin problems and loss of condition in adults and diarrhoea in lambs the highest.

**TABLE 8:** Importance of selected categories of disease problems.

Subcategory	Age group	Farmers		Advisors		Technicians		Veterinarians	
		Mean	Range	Mean	Range	Mean	Range	Mean	Range
Sudden deaths	Lambs	9.1	3–10	7.0	0–10	5.5	0–10	7.4	0–10
	Weaners	9.5	0–10	4.1	0–7	5.2	0–10	6.6	0–10
	Adults	9.3	0–10	4.2	0–7	3.4	0–10	5.0	0–10
Itching or wool loss or skin problem	Lambs	9.0	2–10	4.3	2–9	3.6	0–10	4.3	0–10
	Weaners	9.2	3–10	5.0	3–8	4.5	0–10	6.2	0–10
	Adults	9.2	3–10	6.7	5–9	6.9	1–10	7.2	2–10
Diarrhoea	Lambs	8.9	3–10	6.7	5–8	6.6	1–10	7.7	0–10
	Weaners	9.1	0–10	4.3	1–6	5.4	0–10	6.5	0–10
	Adults	8.9	0–10	4.6	1–7	4.1	0–10	3.7	0–9
Losing body condition	Lambs	9.6	5–10	3.8	0–6	4.7	0–9	6.7	0–10
	Weaners	9.3	0–10	4.7	0–6	5.6	0–10	7.3	2–10
	Adults	9.4	5–10	5.9	3–9	6.0	0–10	6.4	0–10
Bloating or swollen belly	Lambs	9.3	0–10	3.2	0–6	4.3	0–10	3.9	0–10
	Weaners	9.3	0–10	3.7	0–7	3.8	0–10	3.7	0–10
	Adults	9.3	0–10	3.6	0–5	4.1	0–10	4.1	0–10
Nervous symptoms	Lambs	9.2	0–10	3.0	0–7	3.5	0–10	4.5	0–10
	Weaners	9.2	0–10	4.1	0–7	4.6	0–10	5.9	0–10
	Adults	9.3	0–10	3.6	0–7	4.2	0–10	6.0	0–10
Not eating	Lambs	9.9	7–10	2.9	0–5	4.2	0–10	5.5	0–10
	Weaners	9.9	7–10	2.8	0–5	3.5	0–9	5.1	0–10
	Adults	9.9	7–10	2.9	0–6	4.0	0–10	4.8	0–10
Lambing problems	Lambs	9.9	7–10	2.9	0–6	2.2	0–10	0.3	0–2
	Weaners	9.6	0–10	2.4	0–5	2.2	0–10	0.4	0–4
	Adults	9.4	0–10	5.2	0–10	4.4	0–10	5.0	0–10
Blindness	Lambs	9.3	0–10	3.6	0–9	2.0	0–6	1.7	0–9
	Weaners	9.3	0–10	3.9	1–6	2.1	0–6	2.8	0–9
	Adults	8.6	0–10	4.3	3–7	3.7	0–10	3.6	0–10
Others (specify type of problems)	Lambs	2.8	0–10	0.0	0–0	0.0	0–0	0.0	0–0
	Weaners	2.6	0–10	0.0	0–0	0.0	0–0	0.0	0–0
	Adults	2.7	0–10	0.0	0–0	0.0	0–0	0.2	0–4

In answer to questions on measures to manage the most important conditions, the three technical groups cited appropriate measures for the problems. The farmers’ responses indicated that the most popular measures were vaccination and treatments with tetracyclines, penicillin or macrocyclic lactones. However, these were often used for the wrong condition or alone when other remedies would have been better. Examples were the use of penicillin for heartwater and tetracyclines for pulpy kidney and bluetongue.

The main challenges identified for controlling diseases are summarised in Table 9. Again, the farmers awarded high scores to all the issues, with lack of information receiving the highest. The technicians and veterinarians awarded the highest score to lack of farmer training and the advisors rated lack of expert assistance the highest. Collectively, the answers indicate that lack of knowledge and skills and inability to access them are the most important constraints for controlling diseases in small ruminants.

**TABLE 9:** Main problems in controlling diseases.

Subcategory	Farmers		Advisors		Technicians		Veterinarians	
	Mean	Range	Mean	Range	Mean	Range	Mean	Range
Lack of information for farmers	9.7	5–10	7.1	6–8	5.7	2–10	7.5	0–10
Lack of training for farmers	9.2	2–10	6.9	4–9	6.5	2–10	8.8	5–10
Lack of expert assistance	8.8	0–10	7.3	4–9	4.6	0–10	5.7	0–10
Lack of remedy and drug availability	7.7	0–10	7.0	3–9	5.5	0–10	6.4	1–10
Dosage or pack size	8.7	3–10	6.3	4–10	5.0	0–10	5.5	0–10
Cold chain	8.7	0–10	7.2	4–10	4.7	0–9	6.0	0–10
Disease alerts lacking	8.6	0–10	5.9	4–8	4.8	0–10	5.9	0–10
Others	0.4	0–10	0.0	0–0	1.0	0–10	1.4	0–10

Table 10 shows the results for solutions to problems identified with more than 10% support by the farmers and the technical groups. The remaining solutions proposed by the farmers received support from fewer than 5% of the farmers and other proposals from technical groups received less than 6% support. If the farmers’ responses are grouped, then drugs, treatments and vaccines come out as the biggest cluster (45.4%) followed by training (32.7%) and veterinary assistance (12.7%). Among the service providers, a cluster of aspects on farmer training was biggest at 55.8%, followed by drug-related aspects (33.0%) and then actions to be taken by provincial veterinary authorities (18.1%).

**TABLE 10:** Solutions that received support from more than 10% of respondents.

Solution	Region				Groups			Total	%
	20	21	23	24	A	T	V		
**Farmers**									
Financial assistance or vouchers to purchase drugs	4	6	5	4	-	-	-	19	20.0
Disease identification training	6	3	4	2	-	-	-	15	15.8
Dosing programme distribution	1	3	5	5	-	-	-	14	14.7
Discount on medicines and vaccines	3	5	1	4	-	-	-	13	13.7
Mobile vet clinic visitations	-	1	6	3	-	-	-	10	10.5
**Technical groups**									
Farmer training and information	-	-	-	-	6	19	21	46	33.8
More animal health technicians/staff and expert skills	-	-	-	-	5	6	4	15	11.0
Funding for drugs, equipment and training	-	-	-	-	1	6	7	14	10.3

Table 11 reflects how respondents from the farmer and technical groups rated a list of training pamphlets. Farmer average ratings were between 8.5 and 9.5 out of a possible 10, indicating that there was a demand for all of the information offered. Production advisors gave the highest ratings to material on preventing lamb and kid losses, disease recognition and control, record keeping and veld management. Animal health technicians and veterinarians rated preventing lamb and kid losses as most important, followed by worms in the case of technicians and disease recognition and control and internal and external parasites in the case of veterinarians.

Major concerns not related directly to animal diseases included stock theft, veld management and nutrition and the provision and maintenance of facilities for farmers (Table 12). Farmers also rated marketing issues as highly important.

**TABLE 11:** Value rating of educational material.

Subcategory: Training material needed	Farmers		Advisors		Technicians		Veterinarians	
	Mean	Range	Mean	Range	Mean	Range	Mean	Range
Coping with emergencies	9.0	0–10	7.1	4–10	6.6	0–10	5.4	0–10
Equipment needed for sheep and goat farmers	9.0	2–10	7.7	4–10	6.5	0–10	6.0	0–10
Animal handling and management	8.8	0–10	6.8	4–10	7.1	0–10	7.7	0–10
Preventing lamb and kid losses	9.0	0–10	8.7	7–10	8.1	3–10	9.0	6–10
Clinical examination	9.5	4–10	7.4	5–10	6.5	0–10	6.0	0–10
Postmortem technique	9.4	5–10	7.6	5–10	5.3	0–10	4.6	0–10
Samples and specimens	9.6	0–10	5.6	0–10	6.4	0–10	4.9	0–10
Internal parasite control	9.2	0–10	8.0	5–10	8.0	310	8.5	3–10
External parasite control	8.9	0–10	7.8	5–10	7.4	0–10	8.3	4–10
Buying animals safely	9.2	0–10	8.2	6–10	6.9	0–10	7.8	2–10
Disease recognition and control	8.7	0–10	8.7	7–10	7.4	1–10	8.8	4–10
Record keeping	8.9	0–10	8.7	6–10	6.7	0–10	7.6	2–10
Feeding and supplements	8.7	0–10	7.9	6–10	7.2	0–10	8.1	0–10
Breeding methods	8.5	0–10	7.2	4–8	6.7	0–10	7.2	0–10
Selection and culling	9.3	0–10	6.2	4–8	6.7	0–10	7.4	0–10
Vaccines and immunity	9.0	0–10	8.1	5–10	7.3	0–10	8.0	0–10
Treatment methods	9.2	0–10	7.6	6–10	7.1	0–10	7.0	0–10
am management	9.1	0–10	7.9	5–10	6.6	0–10	7.1	0–10
Veld management	9.5	0–10	8.8	7–10	7.7	0–10	7.9	0–10

**TABLE 12:** Ranking of problems and solutions.

Item	Ranking of problem (1–6)				
	Farmers	Advisors	Technicians	Veterinarians	Mean
Farmer training	4	3	1	1	2.25
Veld management	2	2	3	2	2.25
Fences	1	1	4	4	2.25
Budget	2	-	5	3	3.33
Farmer facilities	-	6	2	6	4.66
Marketing	5	-	-	-	5.00
Stock theft and facilities	7	3	5	-	5.00
Predators or dogs	6	-	-	-	6.00

Unlisted information requested by a majority of farmers involved instruction material for shearing and wool sorting. Production advisors emphasised the need for information or training on animal identification, shearing, genetic improvement and improvement of infrastructure. Animal health technicians wanted material for animal identification, as well as selection and culling, farming as a business, marketing of produce, disease recognition, disposal of carcasses, land tenure, facilities, movement of animals and advanced techniques like artificial insemination and embryo transfer. Veterinarians wanted farmer courses or training manuals for feed supplementation, marketing of produce, ram testing, farming as a business, nutrition, slaughter, zoonoses and udder hygiene.

## Discussion

The results of the questionnaire survey revealed that farming communities rated agriculture and livestock as being of high importance, with the emphasis on ruminant livestock, indicating these are important for income generation. However, both the farmers and their service providers (production advisors, animal health technicians and veterinarians) identified numerous constraints that prevent livestock production from reaching its full potential in terms of income generation and wealth creation. The farmers reported experiencing many animal health problems but these were to a large extent dwarfed by larger problems, many of them related to land use, management and availability of products and services that can directly affect the well-being of the animals and the income of farmers. The survey revealed a lack of capacity on the part of the farmers to deal with the problems that could partly be overcome by access to training, information and expert assistance. Although not clearly articulated by farmers, the other groups all rated education, information and training as the most pressing issue.

Although predation is rated by commercial farmers in the rest of South Africa as an enormous problem (Van Niekerk 2010), it was only mentioned by one community and was clearly not seen as an important problem for the overwhelming majority of areas and groups surveyed. This is a significant contrast to other areas and warrants investigation. It is possible that concerns about theft lead to careful night-time penning, thus protecting livestock from predators too, which may have lessons for predator control elsewhere.

The issues relating to veld management and fences contribute to the problem of nutrition, as communal lands are usually permanently over-grazed. The system of communal land ownership is inimical to sustained and judicious land use, as every farmer will try to maximise use by maximising livestock numbers, to the detriment of the primary resource as well as to livestock. The farming communities that responded to the questionnaire did not perceive nutrition as a major problem but it was rated very highly by all three technical groups, who understood that malnutrition was most likely an important contributor to most if not all the disease problems experienced.

Fencing is related to both stock theft and veld management. Facilities and their maintenance are problems requiring attention. They are obviously needed to enable efficient livestock production, but this is linked to training to enable farmers to benefit fully from them.

The provincial and staff budgets were rated high by all groups except production advisors. This is probably explained by a lack of involvement of that group in budgets of the provincial government. Virtually all farmers wanted over 50% of the provincial agriculture budget spent on overcoming the constraints identified and production advisors agreed with them, but this is unrealistic in overall terms. Most animal health technicians recommended 30% – 50% more often than > 50%, while veterinarians were split between 20% – 30% and 30% – 50% for most responses. These last two categories were probably based on more realistic expectations and knowledge of other priorities in the department.

The low rating of marketing as a key issue except for the farmer group may have been because of the small inputs and insights that the other groups had into the vital role that marketing plays in determining financial success or failure. However, both animal health technicians and veterinarians recognised the need for information on marketing and farming as a business when asked to identify topics for educational material.

As could be predicted, livestock owners rated the importance of diseases in their livestock higher than other groups, but all rated importance of diseases highly (averages 70% – 100%). This supported the contention that animal disease control requires significant continued support. Lack of access to veterinary support, including drugs and vaccines, was identified as a major problem. However, it emerged that many of the farmers did access vaccines and medicines but were not informed about their use and therefore often used them inappropriately or incorrectly. Ways to improve veterinary service delivery to communal farmers need to be explored with the help of pharmaceutical companies and other private sector veterinary service providers.

External parasites, in particular sheep scab caused by the mite *Psoroptes communis ovis*, and internal parasites were identified as highly important diseases of small stock. All categories of respondents consistently rated the control of sheep scab as the biggest area of concern. In spite of the fact that it is a controlled disease in South Africa and compulsory control measures have been in force since 1885 with continued efforts to improve their success, control remains problematic, compounded by the fact that alternatives to chemical control need to be sought because of concerns about ecological impacts, toxicity to humans and development of resistance (Bezuidenhout 2011).

Concerns were also raised about the control of red lice, ticks and other ectoparasites. Effective control of ticks is useful in preventing heartwater, caused by *Ehrlichia ruminantium*, which also emerged as a disease of considerable importance. Vaccination presently requires the use of a blood vaccine that contains live organisms; therefore, animals must be observed frequently and treated for signs of infection. Improved vaccines are under development but are not yet commercially available (Allsopp 2015). Vaccines are also available for the other highly rated diseases, namely pulpy kidney (enterotoxaemia) and related clostridial diseases and bluetongue. The live attenuated bluetongue vaccine used in South Africa is highly effective but onerous to use as several vaccinations are required in order to deliver the large number of strains (OIE 2014). An inactivated vaccine was developed for use against the serotype 8 virus that invaded Europe in 2006 but a monovalent vaccine would be unlikely to provide sufficient protection in South Africa, where multiple strains circulate, although cross-protection among strains occurs (OIE 2014).

Systems for sustainable control of internal parasites were already in place in the province and the indications were that they were working. However, the fact that some farmers identified ‘gid’ (*Coenurus cerebralis*) as important indicated the need for regular treatment of dogs for tapeworms and education to prevent the feeding of raw offal including sheep brains to dogs.

It is probable that the diseases rated as most important can be reliably accepted to reflect the true situation. However, the ratings by farmers of syndromes in relation to age reflected in Table 8 should be interpreted with caution or possibly rejected altogether except where they agree with the opinions of the technical groups. It is, for example, highly improbable that blindness in all the age groups would be more important than diarrhoea in lambs.

All groups saw reproduction and the number of lambs or kids born as the most important production factor, with farmers giving this the highest rating and technicians the lowest. Animal sales were less highly rated, in spite of meat sales being the highest source of income for farmers, including wool farmers. Clearly, some education for all categories is needed. The amount and quality of wool was seen as a higher value by farmers, technicians and advisors but not (by a small margin) by veterinarians. Unless all those involved understand that meat sales (live animals) contribute the biggest proportion of income, the true importance of successful reproduction is lost.

Lack of information and training for farmers is a major category of concern that can relatively easily be addressed by a sustained, coordinated programme, based on the training material provided in a full report submitted to the Wellcome Foundation (G.F. Bath, 2013, unpublished report). It was quite clear that most farmers did not know when vaccination was needed, how often to vaccinate or which animals to target. Similarly, drugs used by farmers were all too often completely wrong or ineffective. Training and education are clearly required to rectify these mistakes.

## Conclusion

Some of the responses were inconsistent or illogical; nevertheless, important observations and opinions emerged that reflect serious constraints for communal farmers in the Eastern Cape province of South Africa. These should be noted, discussed, analysed and acted on by all organisations involved to improve the livestock situation in the Eastern Cape province communal areas. These findings may have value in other areas not surveyed in this study.

## Appendix 1

### Question A

On a scale of 0–10 (0 = no importance, 10 = highest importance), how do you view the importance of the following (agriculture in general, all forms of livestock, cattle, sheep, goats, other [chickens, pigs]) to your standard of living?^1^

### Question B

In your view, what are the most important constraints and difficulties that prevent achieving the best results from stock farming? (Open-ended)

### Question C

Which of the following (budget Provincial Department, staff Provincial Department, facilities for farmers, training of farmers, drug supply chain, marketing chain, fences, veld management, stock theft, transport allocations, predators or dogs, animal identification, other [specify]) do you regard as the most important problems that constrain profitable sheep and goat farming? (Score 0–10)

### Question D

What percentage of the annual provincial budget should be spent in overcoming these constraints?

### Question E

On a scale of 0–10 (0 = no importance, 10 = maximum importance), how do you rate the importance of the following categories of all livestock diseases (external parasites, internal parasites, infections, toxic plants, nutrition, other [specify]) in your area of operations?

### Question F

List and prioritise the main problems you encounter and experience in controlling the major diseases of all livestock in your area (0 = no problem, 10 = worst problem). (A 3-column open table with the headings Major Disease, Main Problem and Rating supplied).

### Question G

On a scale of 0–10, how do you regard the importance of the following (reproduction or number of lambs or kids born, number of sheep sold per year, number of goats sold per year, amount of wool sold per year, other [specify])?

### Question H

On a scale of 0–10 (0 = no importance, 10 = highest importance), how do you rate the importance of diseases to sheep and goat farming in your area?

### Question I

On a scale of 0–10, how do you regard the importance of the following disease problems (sudden deaths, itching or wool loss or skin problem, diarrhoea, losing body condition, bloating or swollen belly, nervous symptoms, not eating, lambing problems, blindness, others [specify type of problem]) in sheep and/or goats?

### Question J

Name or specify the most important disease conditions which you experience in goats and/or sheep in your area? Rate them as high (H), medium (M) or lower (L) priority. (A 2-column open table with the headings Disease and Priority follows).

### Question K

Do you have solutions (e.g. treatment, vaccines, management options) to overcome these major problems, and if so what are they (list per problem)? In your opinion, how well do they work? (0–10 scale, 0 = not at all, 10 = perfectly). (A 3-column open table with the headings Disease [name of problem], Solution used – Treatments, vaccines, management, etc. and Rating of success [0–10, worst to best] follows).

### Question L

On a scale of 0–10, what are the main problems you experience in controlling diseases in general in your area (sheep and goat farms)? (Options: lack of information for farmers, lack of training for farmers, lack of expert assistance, lack of remedy and drug availability, cost of drugs or equipment, dosage or pack size, cold chain, disease alerts lacking; others could be added).

### Question M

Please give your suggestions to solve these problems (followed by an open block to enable respondents to provide answer to open-ended question).

### Question N

On a scale of 0–10, how do you rate the following proposed educational pamphlets or training sessions for sheep and goat farmers? (Coping with emergencies, equipment needed for sheep and goat farmers, animal handling and management, preventing lamb and kid losses, clinical examination, postmortem technique, samples and specimens, internal parasites control, external parasites control, buying animals safety, disease recognition and control, record keeping, feeding and supplements, breeding methods, selection and culling, vaccines and immunity, treatment methods, ram management and veld management).

### Question O

Apart from the list of proposed training and educational material listed above (Question N), are there other items you believe should be added? (A 2-column open table with headings Problem or training needed and Rating followed).
